# Axenic *Leishmania amazonensis* Promastigotes Sense both the External and Internal Arginine Pool Distinctly Regulating the Two Transporter-Coding Genes

**DOI:** 10.1371/journal.pone.0027818

**Published:** 2011-11-16

**Authors:** Emerson A. Castilho-Martins, Maria Fernanda Laranjeira da Silva, Marcos G. dos Santos, Sandra M. Muxel, Lucile M. Floeter-Winter

**Affiliations:** Departamento de Fisiologia, Instituto de Biociências, Universidade de São Paulo São Paulo, Brazil; The University of Maryland, United States of America

## Abstract

*Leishmania (L.) amazonensis* uses arginine to synthesize polyamines to support its growth and survival. Here we describe the presence of two gene copies, arranged in tandem, that code for the arginine transporter. Both copies show similar Open Reading Frames (ORFs), which are 93% similar to the *L. (L.) donovani AAP3* gene, but their 5′ and 3′ UTR's have distinct regions. According to quantitative RT-PCR, the 5.1 *AAP3* mRNA amount was increased more than 3 times that of the 4.7 *AAP3* mRNA along the promastigote growth curve. Nutrient deprivation for 4 hours and then supplemented or not with arginine (400 µM) resulted in similar 4.7 *AAP3* mRNA copy-numbers compared to the starved and control parasites. Conversely, the 5.1 *AAP3* mRNA copy-numbers increased in the starved parasites but not in ones supplemented with arginine (p<0.05). These results correlate with increases in amino acid uptake. Both Meta1 and arginase mRNAs remained constant with or without supplementation. The same starvation experiment was performed using a *L. (L.) amazonensis* null knockout for arginase (*arg* ^-^) and two other mutants containing the arginase ORF with (*arg* ^-^/ARG) or without the glycosomal addressing signal (*arg* ^-^/*arg*ΔSKL). The *arg* ^-^ and the *arg* ^-^/*arg*ΔSKL mutants did not show the same behavior as the wild-type (WT) parasite or the *arg* ^-^/ARG mutant. This can be an indicative that the internal pool of arginine is also important for controlling transporter expression and function. By inhibiting mRNA transcription or/and mRNA maturation, we showed that the 5.1 *AAP3* mRNA did not decay after 180 min, but the 4.7 *AAP3* mRNA presented a half-life decay of 32.6 +/− 5.0 min. In conclusion, parasites can regulate amino acid uptake by increasing the amount of transporter-coding mRNA, possibly by regulating the mRNA half-life in an environment where the amino acid is not present or is in low amounts.

## Introduction

Leishmaniasis is a complex parasitic disease that currently affects about 12 million people and an estimated 2 million new cases per year [1]. It is caused by protozoa in the *Leishmania* genus, which has two distinct phases in its life cycle: the promastigote, an extracellular flagellate present at the gut of sand flies, and the amastigote that lives inside mononuclear phagocytes, mainly macrophages, in a vertebrate host.

Arginine is a key amino acid for macrophages because, being the substrate for inducible nitric oxide synthase (iNOS) to produce nitric oxide (NO), it is involved in the macrophage-defense response against pathogen infections. [2]–[8]. This amino acid is also a substrate for arginase, which catalyzes the production of urea and ornithine, a product important for polyamine pathway. This pathway is used by *Leishmania* to replicate and is essential for the parasite to establish infection [9]–[12]. It has largely been reported that macrophage or *Leishmania* modulation of arginine is responsible for parasite survival or its killing in the mammal host [5], [13]–[19].

Membrane transporters, present in both *Leishmania* and macrophages control arginine uptake [20]–[24], to sustain NO production, macrophages increase their expression of the main arginine transporter (CAT2B), which is indicative that the internal pool of arginine is not sufficient to supply arginine to iNOS [25]–[27]. On the other hand, a high-affinity arginine transporter has been described in *L. (L.) donovani*. This transporter is LdAAP3, and it has 480 amino acids and 11 predicted trans-membrane domains [22]. With this transporter, *Leishmania* seems to have mechanisms of sensing arginine decreases and responding with increased arginine uptake [28]. Therefore, the arginine-uptake control appears to be an important limiting factor to parasite survival inside macrophages [17], [29].

*Leishmania* has a polycistronic transcription, and the control of gene expression is mainly performed through protein levels and mRNA stability [30]. In this study, we evaluated the importance of arginine transporter mRNA levels on the physiology of arginine uptake in *L. (L.) amazonensis*. Our data indicated that these organisms control the arginine transporter expression by regulating the transporter-coding mRNA stability. We also showed that the level of arginine transporter mRNA varies in promastigote development, and, using arginase-deficient mutants, we showed that possible changes in the internal arginine pool could be responsible for altering the transporter-coding mRNA levels.

## Results

### Characterization of the two L. (L.) amazonensis arginine transporter-coding DNA sequences

A DNA probe based on the *AAP3* ORF sequence of *L. donovani* [22] was used to screen a *L. (L.) amazonensis* genomic-cosmid DNA library [31]. The partial DNA sequence of the selected cosmid revealed the presence of two copies in tandem from a putative homologous gene. The ORF regions of the two copies showed 93% similarity to the *AAP3* ORF in *L. donovani* (not shown). A northern blot analysis showed the presence of two distinct mRNAs for the gene (5.1 kb and 4.7 kb) (not shown). We named these transcripts 4.7 *AAP3* mRNA and 5.1 *AAP3* mRNA. The mRNA identities were confirmed by sequencing RT-PCR products that were obtained using oligo-dT reverse transcription and primers based on the cosmid sequence. These sequences were deposited in GenBank with accession numbers of HQ912026 (5.1 *AAP3*) and HQ912027 (4.7 *AAP3*).

The ORFs of each copy, that are 98% identic were clone into pYES2 plasmid and the recombinant plasmids were used to transform a *Saccharomyces cerevisiae* mutant lacking amino-acid transporter coding region: GAP1/ YHR039W (Material and Methods S1). The transformed yeast recovered the growth capacity in medium containing L-arginine confirming the transporter character of the protein encoded by those sequences (Figure S1).

The two transcribed copies of the gene presented different 5′ untranslated regions (5′UTRs). This allowed for the design of specific primers to differentially quantify each copy by quantitative reverse transcription PCR (qRT-PCR). The alignment of the 5′UTRs from the two copies, their differences and primers positions are shown in Figure S2.

### Arginine uptake by L. (L.) amazonensis promastigotes correlates with the arginine transporter transcript abundance

**Figure 1:**
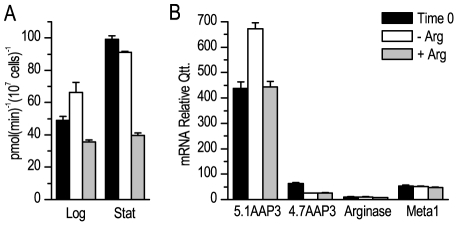
Amino acid starvation regulates the arginine transporter rate and 5.1 kb *AAP3* mRNA level in *L. (L.) amazonensis*. A. Arginine uptake of mid-log and stationary phase *L. (L.) amazonensis* in non-starved parasites (black), 4-h starved parasites (white) and 4-h starved + 400 µM arginine parasites (gray). B. Total RNA of non-starved parasites (black), 4-h starved parasites (white) and 4-h starved + 400 µM arginine parasites (gray). RNA was used to prepare the cDNA, as described in Material and Methods. Equal amounts of cDNA were then used in qRT-PCR to determine the copy-number of both copies of the arginine transporter, arginase and Meta1. All determinations were normalized by GAPDH. Results of a representative experiment. Data are shown as the mean±S.E. (n = 3).

It is known that *L. donovani* promastigotes are sensitive to arginine starvation, and they respond with an increase in both arginine transporter expression and transport rate [28]. However, it is not completely clear at which level of protein-expression regulation this control occurs. We performed an arginine starvation on mid-log and stationary *L. (L.) amazonensis* promastigotes for 4 h at 25°C and then evaluated arginine uptake. Initially, we could note that at time 0 that means the physiological condition in each phase the arginine uptake in mid-log phase parasites is lower than the one detected in the stationary phase parasites. When starved, the mid-log phase parasites showed an increase in the arginine uptake compared to the control parasites at time 0 (p<0.05). As this behavior was not detected in starved stationary phase parasites, we can conclude that stationary phase parasites do not respond to starvation (Figure 1A). However, the increase in arginine uptake was not observed when the mid-log phase parasites were incubated for the same time in the presence of arginine (400 µM) (Figure 1A), but the arginine uptake of the stationary phase parasites decrease, in relation to time 0, when incubated in the presence of arginine (Figure 1A). We also evaluated mRNA level at mid-log parasites, and the increase of arginine uptake correlated with an increase in the relative copy-number of the 5.1 *AAP3* mRNA (Figure 1B, p<0.05), suggesting the existence of at least one pre-translational mechanism for controlling protein expression. Moreover, only the 5.1 *AAP3* mRNA was sensitive to the amino acid starvation. No differences were observed in the 4.7 *AAP3* mRNA copy-number or the mRNAs coding for arginase and Meta1, all normalized by the GAPDH mRNA copy-number (Figure 1B).

**Figure 2:**
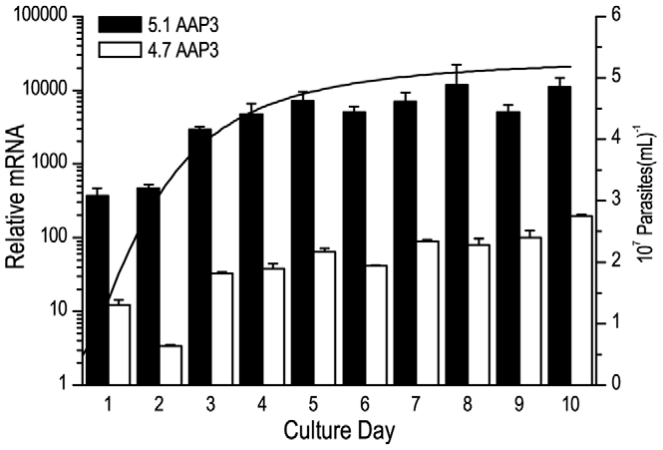
mRNA quantification from the parasite culture-growth curve. Total RNA from each day on the culture growth curve was used to obtain the cDNA. mRNA of both arginine transporters in *L. (L.) amazonensis* were determined (5.1 *AAP3* mRNA in black; 4.7 *AAP3* mRNA in white), and both were normalized to GAPDH mRNA. The black line represents the parasites' growth curve. The results of a representative experiment. Data are shown as the mean±S.E. (n = 4).

To evaluate differences in the amino acid-starvation response between mid-log and stationary parasites, we determined the amount of *AAP3* mRNA from the 1^st^ to the 10^th^ day of a culture growth curve as described in methods. We compared the mRNA expression amounts of the 5.1 *AAP3* mRNA and the 4.7 *AAP3* mRNA normalized to GAPDH mRNA. The arginine transporter mRNA was increased more than 10 times in the stationary parasites compared to the log-phase parasites (Figure 2). Interestingly, both copies increased the mRNA level in the stationary phase, although the 5.1 *AAP3* mRNA was at least 30 fold more abundant than the 4.7 *AAP3* mRNA at the log-phase, reaching 100 fold in the stationary phase.

### Arginine-transporter-coding transcripts stability are affected in axenic promastigotes experiencing arginine deprivation

**Figure 3:**
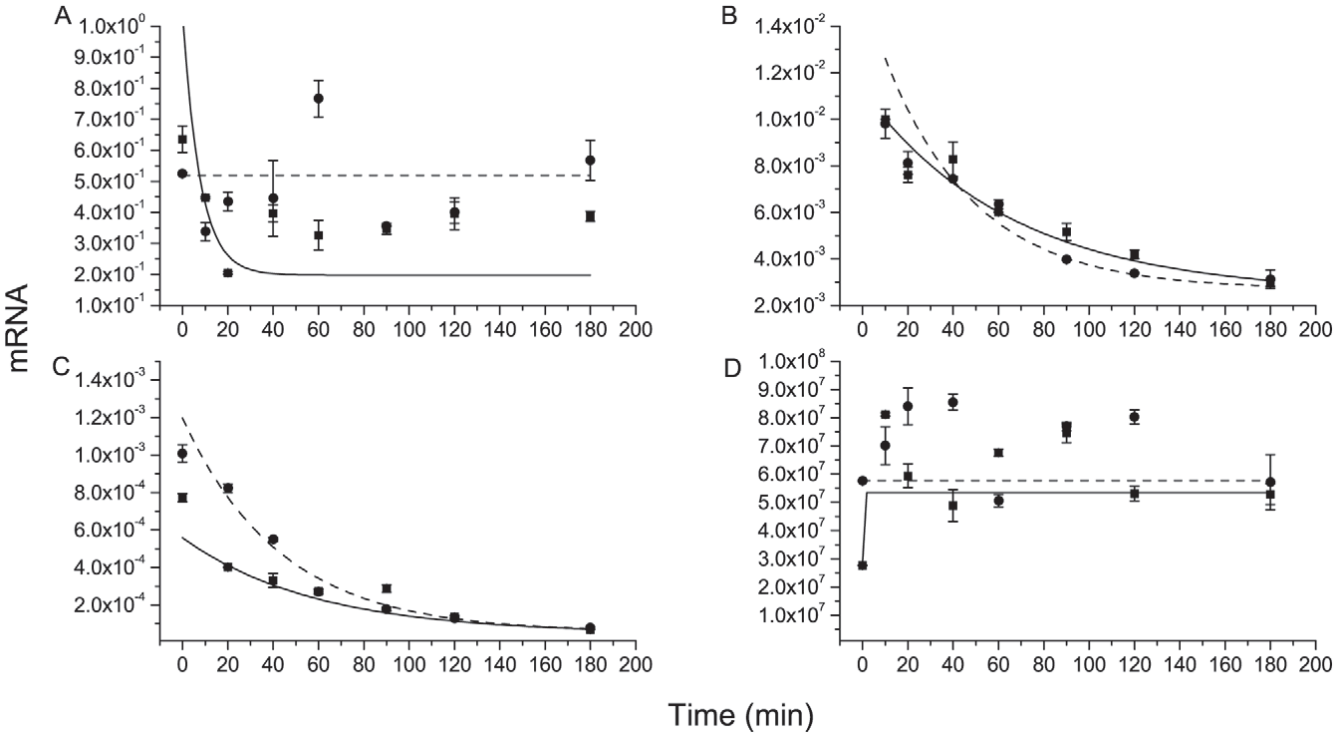
mRNA decay of *L. (L.) amazonensis* total mRNA. Relative copy-numbers of: A. 5.1 *AAP3* mRNA transporter, B. 4.7 *AAP3* mRNA transporter, C. GAPDH mRNA and D. SSUrRNA. A, B and C were normalized by SSUrRNA. The black line represents the exponential decay fit of actinomycin + sinefungin-treated samples in presence of 400 µM arginine. The discontinuous line represents the exponential decay fit of actinomycin + sinefungin-treated samples in absence of arginine. Fit lines for B and C have R^2^>0.90. Data of a representative experiment shown as the mean±S.E. (n = 3).

Treatment with actinomycin and sinefungin causes an inhibition of transcription and *trans-*splicing mRNA-maturation processes in the parasites [32], [33]. RNA obtained from a time-course treatment of mid-log phase promastigotes with actinomycin D and sinefungin, preceded or not by 4 hours arginine starvation was used in qRT-PCR experiments, as described in Material and Methods. The 5.1 *AAP3* mRNA showed no decay after 180 min of treatment in cells submitted to arginine starvation but the presence of the amino acid induces a degradation (Figure 3A), in contrast to the observed for both 4.7 *AAP3* mRNA [half lives of 45.7±4.5 min (+arg)/ 27.7±5.4 (-arg)] and GAPDH [(half lives of 40.6±2.1 min (+arg)/ 30.0±5.4 (-arg)] (Figure 3B and 3C). The qRT-PCR data were normalized by SSUrRNA copy-number, a RNA that is not sensitive to the inhibitor drugs (Figure 3D).

### The arginine transporter transcript abundance also assesses the sensing of the arginine internal pool: an output relying on L. amazonensis genetic manipulation targerting the arginase-coding gene

**Figure 4:**
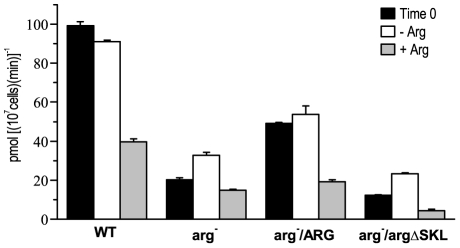
Arginine uptake in *L. (L.) amazonensis*. Promastigotes from wild-type (WT), arginase knockout mutant (*arg* ^-^), arginase genetically-complemented mutant with glycosomal addressing signal (*arg^-^*/ARG) and arginase-complemented mutant without the glycosomal addressing signal (*arg* ^-^/*arg*ΔSKL) were treated with labeled arginine for 5 min to determine the arginine uptake, as described in the Material and Methods. Bars represent non-starved parasites (black), 4-h starved parasites (white) and 4-h starved + 400 µM arginine parasites (gray). Data of a representative experiment shown as the mean±S.E. (n = 3).

To evaluate the influence of an internal pool of arginine on its uptake, we used a *L. (L.) amazonensis* arginase-null mutant (*arg* ^-^), which does not use arginine to produce ornithine and present higher amounts of arginine in their cytoplasm, requiring polyamines supplementation (Laranjeira da Silva, submitted). The mutant and WT parasites (5×10^7^/mL), at initial stationary phase, were submitted to amino acid starvation and then arginine uptake was evaluated. Although both parasites responded to amino acid starvation, the WT parasites presented greater arginine uptake than the *arg* ^-^ (Figure 4). We performed the same assay using a knockout mutant that is genetically complemented with the arginase ORF (*arg* ^-^/ARG) showing a partial recovery in arginase activity (Laranjeira da Silva, submitted). Interestingly, this mutant also presented a partial recovery in arginine uptake, compared to the WT. On the other hand, the complemented mutant, that contained the arginase ORF without the correct glycosomal compartmentalization signal (*arg* ^-^/*arg*ΔSKL) and did not present any arginase activity (Laranjeira da Silva, submitted), showed a arginine uptake similar to the *arg* ^-^ mutant (Figure 4).

## Discussion

The data presented in this study show that *L. (L.) amazonensis* can control arginine uptake when promastigotes parasites are amino acid starved. These observations are similar to those made in *L. donovani* [28]. Adding to that data, we showed that the internal pool of the amino acid is also important to regulate the uptake. Furthermore, we showed that the higher concentration of one of the *AAP3* transcripts is due to a stabilization process in the mature mRNA and not to an increase in the transcriptional rate or mRNA *trans*-splicing maturation. Most eukaryotes generally control their gene expression at the transcriptional level; however, *Leishmania* lacks this control mechanism because its transcription is polycistronic [34]. Besides, there are no known RNA polymerase II promoter regions that have binding sites for transcriptional regulatory factors. However, these organisms can control gene expression at the mRNA maturation level (poly-adenylation/*trans*-splicing coupled processes) or by changing mRNA half-lives in different conditions [30]. This digenetic organism experiences different environmental conditions, such as pH, temperature and nutrient availability, when it cycles between invertebrate and mammalian hosts [35]. Our findings suggest a possible mechanism for the parasite to overcome the different requirements due to environmental changes that they will find in insect gut along their development, going from a nutrient rich media after insect blood meal through an deprived ambient until the next feed.

A possible physiological explanation for the presence of two copies of the *AAP3* gene is that each copy could be differentially regulated according to the environmental conditions of the parasite's differentiated stage. It is interesting that one of the arginine transporter mRNAs (4.7 *AAP3* mRNA) presented the same typical decay behavior observed for GAPDH mRNA when promastigotes were treated with actinomycin and sinefungin. The other transcript (5.1 *AAP3* mRNA) remains stable even 180 min after blocking of transcription and *trans*-splicing. This could explain the reason why this copy presents at least 30-fold more copies than the 4.7 *AAP3* mRNA. Previous reports show that the mRNA stability in these organisms is altered by amino acid availability, but the molecular mechanisms by which this occurs are still unclear [36]–[39]. As arginine internal pool is also important in regulating arginine uptake, we may speculate if, in some way, the amino acid concentrations in extra- or intracellular medium could regulate *AAP3* mRNA decay.

It is well known that arginine plays a critical role as substrate for macrophages to kill *Leishmania* via iNOS activity [19], but macrophage's arginine internal pool is not enough to provide substrate for either Th1 or Th2 responses [40], highlighting the importance of the arginine uptake control. As our results suggests, stationary phase promastigotes can uptake higher amounts of arginine than parasites in log phase. If so, the arginine influx into macrophages may be buffered if recently phagocytized *Leishmania* promastigotes, with increased arginine transport, sequester arginine from the macrophages. This is especially true when considering that macrophage CAT2 has no sensitivity to arginine concentrations [41].

*L. (L.) major* arginase-null mutants show a delay in starting lesions in *in vivo* infections [42]. The *L. (L.) amazonensis* arginase-null mutants also presented a delay in *in vivo* infectivity (Laranjeira da Silva, submitted). Under arginine starvation, the arginine uptake in the *arg* ^-^ mutants was less pronounced than in the WT parasites. This infective-capacity impairment may be attributed to both arginine uptake and/or arginase activity. The uptake pattern of *arg* ^-^ tended to be reverted in the mutant that was genetically complemented with the WT arginase ORF, but the pattern did not revert when the genetic complementation was done with an arginase ORF without the correct glycosomal addressing signal. This supports the idea that arginase exerts some control on arginine uptake and arginase activity, and arginine uptake is crucial to parasite survival inside macrophages. In addition, the lower arginine uptake that occurred in response to starvation in the null mutants indicates a mechanism to increase arginine transporter mRNA. This may occur through detecting possible changes in the internal arginine pool because the disruption of one arginine pathway decreases responses to amino acid starvation, which is also described in *L. donovani* ornithine decarboxylase or spermidine synthase-null mutants [28].

Stationary phase WT parasites have an increased expression of the arginine transporter, in relation to mid-log phase parasites, but did not respond to amino acid starvation. On the other hand, stationary phase *arg* ^-^ mutant responds to arginine starvation like mid-log phase WT parasites (Figure 1A and Figure 4). A possible explanation is that the mutant uses less arginine present in the culture media, keeping a higher concentration compared to the WT growing media. The data suggests that the maximum expression of the transporter may have been already reached at WT stationary phase. Achieving maximum transporter expression may be a response to decreases in nutrient concentrations in the culture medium over time, and it may represent an adaptation to the low-nutrient availability found inside the fly mid-gut after blood digestion was completed. Another mechanism could be that nutrient depletion drives the modifications in promastigote parasites that induce differentiation in the infective stage [43]. Thus, arginine depletion may represent a signal to metacyclogenesis, although we did not observe changes in *Meta1* mRNA due to amino acid starvation. This is possibly because we only observed them for 4 hours.

The results presented in this study lead us to conclude that arginine uptake is controlled by transporter-coding mRNA levels in *L. (L.) amazonensis.* They also suggest a mechanism that senses internal arginine concentrations and controls arginine uptake by increasing arginine transporter expression. This may represent a part of metacyclogenesis for achieving the infective stage.

## Materials and Methods

### Organisms

Wild-type (WT) promastigotes from the *L. (L.) amazonensis* strain MHOM/BR/1973/M2269 and three arginase mutants [*arg* ^-^, *arg^-^*/ARG and *arg^-^*/*arg*ΔSKL (Laranjeira da Silva, submitted)] were maintained at 25°C by inoculating 5×10^6^ parasites in M199 medium (10 mL) supplemented with 10% fetal calf serum (FCS-Invitrogen - Carlsbad, USA) in 25 cm^2^ tissue culture flasks. The supplemented media was changed every 7 days. Arginase-null mutants were also supplemented with putrescine (50 µM).

The mRNA expression and arginine uptake were evaluated along the growth curve by maintaining the parasites at log phase by sub-culturing them every 24 h with the same initial cell ratio (5×10^5^ parasites/mL), as previously described [44].

### AAP3 gene cloning

Based on sequences described by Shaked-Mishan et al. [22], we amplified the *L. (L.) donovani AAP3* ORF from genomic DNA, purified as described previously [45]. We used this amplicon as a template to construct a radioactive probe (0.1 mCi). This probe was constructed using α^32^P-dCTP (10 mCi/mL; 3,000 Ci/mmol; GE Healthcare, UK) and Amersham Megaprime DNA Labeling Systems (GE Healthcare, UK) following manufacturer's standard protocol. This probe was used to screen a *L. (L.) amazonensis* cosmid library [31] (kindly provided by S.R. Uliana, ICB-USP), and cosmid DNA was printed from bacterial-containing plates onto a nylon membrane [46]. Hybridization was performed at 42°C overnight followed by two separate, 20-min washes at ambient temperature and 50°C with SSC 2X SDS (0.1%). The nylon membrane was exposed to X-ray film (Kodak) and developed according the manufacturer's protocol. Selected clones were recovered in SOB growth media, and cosmid DNA was isolated by alkaline lysis [45]. Sequencing of the cosmid DNA was performed by the Sanger dideoxy protocol as described previously [47].

### RNA purification, cDNA synthesis and qRT-PCR

RNA was extracted with Trizol Reagent (Invitrogen) using the manufacturer's protocol. Reverse transcription was performed with a random primer protocol (Fermentas, M-MuLV RT) using total RNA (2 µg). The obtained cDNA was diluted in water and used in quantitative Real-Time PCR (qRT-PCR) with primers (Table 1) designed to differentially amplify the 5′UTR region of the two copies of the *AAP3* gene. The expected products were cloned and sequenced to validate the PCR. Known amounts of the cloned-DNA products were calculated (number of molecules), and they were used in the qRT-PCR to produce the standard curve. The following protocol was used in the qRT-PCR: 50 total cycles encompassing an association/fragment extension step at 61°C for 50 s and a denaturation step for 20 s at 94°C. A 7300 System (Applied Biosystems, USA) was used to run the qRT-PCR. The primers used to amplify GAPDH (internal control), arginase and SSUrRNA are described elsewhere [44].

**Table 1:** Primer sequences used in PCR reactions and the amplified products.

Name	Sequence (5′-3′)	Used to
TArg5U1KF	GGT CCC CGA TAC ACA CAT TC	Amplify 5′UTR of 5.1 mRNA
TArg5U1KR	GTC TCC CGT TTT GCA AGA GA	
TArg5u500bF	ACC ATT GTG GGT TAG TTA TAC ATC C	Amplify 5′UTR of 4.7 mRNA
TArg5u500bR	CAA GAT CGC TAG CAG TGG AG	

### Uptake assays

We adapted a protocol from dos Santos et al. [48] for the uptake assays. Briefly, promastigotes in the mid-log phase (2×10^7^ parasites/mL) or in the initial stationary phase (4.5×10^7^) were washed twice with cold Earle's Based Salt Solution (EBSS) (LGC Biotecnologia, SP, Brazil) and resuspended at 2×10^8^ parasites/mL. We combined 50 µL of this mixture (10^7^ parasites) with 50 µL of ^3^H-Arginine (40 µM; 1 µCi/mL; GE Healthcare, UK) at 25°C. Uptake was stopped at different times by adding 50-mM ice-cold arginine (Ajinomoto, Tokio, Japan) (200 µL). Parasites were then washed twice with EBSS (200 µL), and radioactivity was measured by liquid scintillation spectrometry in a 2100 TR Packard Tri-Carb Liquid Scintillation Counter (PerkinElmer, USA).

### Starvation assay

We used a protocol described by Daslyuk et al. [28] to starve the promastigotes, with one difference: the parasites were kept for 4 h at 25°C. Controls were performed at time 0 by putting the parasites on ice or incubating the parasites in the presence of arginine (400 µM).

### Actinomycin and Sinefungin treatments

RNA half-lives were determined using actinomycin D (Sigma-Aldrich, MO, USA) (10 µg/mL) and sinefungin (Sigma-Aldrich, MO, USA ) (2 µg/mL) as described by Stewart & Clayton [32]. At different times, the parasites were placed in EBSS, and the treatments were stopped by lysing the parasites with Trizol Reagent (Invitrogen, USA) for RNA extraction.

### Statistical data analysis

Statistical significance was determined by Student's t test (p<0.05).

## Supporting Information

Material and Methods S1 **Methodological description of genetic complementation of yeast mutant.**
(DOC)

Figure S1 **Genetic complementation of yeast mutant certifies the AAP functional character of** ***L. amazonensis*** **amino acid transporter ORFs.** pYES2 plasmid DNA carrying 5.1 *AAP3* and 4.7 *AAP3* ORFs complemented *Saccaromyces cerevisiae* mutant GAP1/YHR039W. The transformed yeast clones, ORF 4.7 and ORF 5.1, obtained as described in Material and Methods S1 were able to growth in the minimal medium supplemented with 1 mg/m of L-arg (A). The mutant transformed with the recipient plasmid pYES2 alone, only grew in a medium containing ammonium as (NH4+) as nitrogen source (B).
(TIF)

Figure S2 **Sequence alignment of the 5′UTRs from 5.1** ***AAP3*** **mRNA and 4.7** ***AAP3*** **mRNA.** Cyan-colored box represents the *Spliced-Leader* RNA sequence. Gray box represents the ORF beginning. Blue boxes show the primers for amplifying the 5.1 *AAP3* mRNA, and the green boxes show the primers for amplifying the 4.7 *AAP3* mRNA.
(TIF)

## References

[1] Desjeux P (2004). Leishmaniasis: current situation and new perspectives.. Comp Immunol Microbiol Infect Dis.

[2] Murray HW (1982). Cell-mediated immune response in experimental visceral leishmaniasis - II Oxygen- dependent killing of intracellular Leishmania donovani amastigotes.. Journal of Immunology.

[3] Murray HW, Cartelli DM (1983). Killing of intracellular Leishmania donovani by human mononuclear phagocytes. Evidence for oxygen-dependent and -independent leishmanicidal activity.. J Clin Invest.

[4] Murray HW, Szuro-Sudol A, Wellner D, Oca MJ, Granger AM (1989). Role of tryptophan degradation in respiratory burst-independent antimicrobial activity of gamma interferon-stimulated human macrophages.. Infect Immun.

[5] Liew FY, Millott S, Parkinson C, Palmer RM, Moncada S (1990). Macrophage killing of Leishmania parasite in vivo is mediated by nitric oxide from L-arginine.. J Immunol.

[6] Assreuy J, Cunha FQ, Epperlein M, Noronha-Dutra A, O'Donnell CA (1994). Production of nitric oxide and superoxide by activated macrophages and killing of Leishmania major.. Eur J Immunol.

[7] Liew FY, Xu D, Chan WL (1999). Immune effector mechanism in parasitic infections.. Immunol Lett.

[8] Murray HW, Nathan CF (1999). Macrophage microbicidal mechanisms in vivo: reactive nitrogen versus oxygen intermediates in the killing of intracellular visceral Leishmania donovani.. J Exp Med.

[9] Mukhopadhyay R, Madhubala R (1995). Leishmania donovani: cellular control of ornithine decarboxylase in promastigotes.. Int J Biochem Cell Biol.

[10] Fairlamb AH, Cerami A (1992). Metabolism and functions of trypanothione in the Kinetoplastida.. Annu Rev Microbiol.

[11] Yoshida N, Camargo EP (1978). Ureotelism and ammonotelism in trypanosomatids.. J Bacteriol.

[12] Camargo EP, Coelho JA, Moraes G, Figueiredo EN (1978). Trypanosoma spp., Leishmania spp. and Leptomonas spp.: enzymes of ornithine-arginine metabolism.. Exp Parasitol.

[13] Roach TI, Kiderlen AF, Blackwell JM (1991). Role of inorganic nitrogen oxides and tumor necrosis factor alpha in killing Leishmania donovani amastigotes in gamma interferon-lipopolysaccharide-activated macrophages from Lshs and Lshr congenic mouse strains.. Infect Immun.

[14] Evans TG, Reed SS, Hibbs-Jr JB (1996). Nitric oxide production in murine leishmaniasis: correlation of progressive infection with increasing systemic synthesis of nitric oxide.. Am J Trop Med Hyg.

[15] Liew FY, Wei XQ, Proudfoot L (1997). Cytokines and nitric oxide as effector molecules against parasitic infections.. Philos Trans R Soc Lond B Biol Sci.

[16] Iniesta V, Gomez-Nieto LC, Corraliza I (2001). The inhibition of arginase by N(omega)-hydroxy-l-arginine controls the growth of Leishmania inside macrophages.. J Exp Med.

[17] Kropf P, Fuentes JM, Fahnrich E, Arpa L, Herath S (2005). Arginase and polyamine synthesis are key factors in the regulation of experimental leishmaniasis in vivo.. Faseb J.

[18] Gaur U, Roberts SC, Dalvi RP, Corraliza I, Ullman B (2007). An effect of parasite-encoded arginase on the outcome of murine cutaneous leishmaniasis.. J Immunol.

[19] Green SJ, Meltzer MS, Hibbs JB, Nacy CA (1990). Activated macrophages destroy intracellular Leishmania major amastigotes by an L-arginine-dependent killing mechanism.. J Immunol.

[20] Deves R, Boyd CA (1998). Transporters for cationic amino acids in animal cells: discovery, structure, and function.. Physiol Rev.

[21] Closs EI, Lyons CR, Kelly C, Cunningham JM (1993). Characterization of the third member of the MCAT family of cationic amino acid transporters. Identification of a domain that determines the transport properties of the MCAT proteins.. J Biol Chem.

[22] Shaked-Mishan P, Suter-Grotemeyer M, Yoel-Almagor T, Holland N, Zilberstein D (2006). A novel high-affinity arginine transporter from the human parasitic protozoan Leishmania donovani.. Mol Microbiol.

[23] Geraldo MV, Silber AM, Pereira CA, Uliana SR (2005). Characterisation of a developmentally regulated amino acid transporter gene from Leishmania amazonensis.. FEMS Microbiol Lett.

[24] Akerman M, Shaked-Mishan P, Mazareb S, Volpin H, Zilberstein D (2004). Novel motifs in amino acid permease genes from Leishmania.. Biochem Biophys Res Commun.

[25] Nicholson B, Manner CK, Kleeman J, MacLeod CL (2001). Sustained nitric oxide production in macrophages requires the arginine transporter CAT2.. J Biol Chem.

[26] Closs EI, Scheld JS, Sharafi M, Forstermann U (2000). Substrate supply for nitric-oxide synthase in macrophages and endothelial cells: role of cationic amino acid transporters.. Mol Pharmacol.

[27] Bogle RG, Baydoun AR, Pearson JD, Moncada S, Mann GE (1992). L-arginine transport is increased in macrophages generating nitric oxide.. Biochem J.

[28] Darlyuk I, Goldman A, Roberts SC, Ullman B, Rentsch D (2009). Arginine homeostasis and transport in the human pathogen Leishmania donovani.. J Biol Chem.

[29] Naderer T, McConville MJ (2008). The Leishmania-macrophage interaction: a metabolic perspective.. Cell Microbiol.

[30] Flinn HM, Smith DF (1992). Genomic organisation and expression of a differentially-regulated gene family from Leishmania major.. Nucleic Acids Res.

[31] Uliana SR, Goyal N, Freymuller E, Smith DF (1999). Leishmania: overexpression and comparative structural analysis of the stage-regulated meta 1 gene.. Exp Parasitol.

[32] Archer S, Queiroz R, Stewart M, Clayton C (2008). Trypanosomes as a model to investigate mRNA decay pathways.. Methods Enzymol.

[33] McNally KP, Agabian N (1992). Trypanosoma brucei spliced-leader RNA methylations are required for trans splicing in vivo.. Mol Cell Biol.

[34] Button LL, Russell DG, Klein HL, Medina-Acosta E, Karess RE (1989). Genes encoding the major surface glycoprotein in Leishmania are tandemly linked at a single chromosomal locus and are constitutively transcribed.. Mol Biochem Parasitol.

[35] Zilberstein D, Shapira M (1994). The role of pH and temperature in the development of Leishmania parasites.. Annual Reviews in Microbiology.

[36] Bruhat A, Jousse C, Wang XZ, Ron D, Ferrara M (1997). Amino acid limitation induces expression of CHOP, a CCAAT/enhancer binding protein-related gene, at both transcriptional and post-transcriptional levels.. J Biol Chem.

[37] Gong SS, Guerrini L, Basilico C (1991). Regulation of asparagine synthetase gene expression by amino acid starvation.. Mol Cell Biol.

[38] Jousse C, Averous J, Bruhat A, Carraro V, Mordier S (2004). Amino acids as regulators of gene expression: molecular mechanisms.. Biochem Biophys Res Commun.

[39] Roberts SC, Tancer MJ, Polinsky MR, Gibson KM, Heby O (2004). Arginase plays a pivotal role in polyamine precursor metabolism in Leishmania. Characterization of gene deletion mutants.. J Biol Chem.

[40] Yeramian A, Martin L, Arpa L, Bertran J, Soler C (2006). Macrophages require distinct arginine catabolism and transport systems for proliferation and for activation.. Eur J Immunol.

[41] Yeramian A, Martin L, Serrat N, Arpa L, Soler C (2006). Arginine transport via cationic amino acid transporter 2 plays a critical regulatory role in classical or alternative activation of macrophages.. J Immunol.

[42] Muleme HM, Reguera RM, Berard A, Azinwi R, Jia P (2009). Infection with arginase-deficient Leishmania major reveals a parasite number-dependent and cytokine-independent regulation of host cellular arginase activity and disease pathogenesis.. J Immunol.

[43] Sacks DL, Perkins PV (1985). Development of infective stage Leishmania promastigotes within phlebotomine sand flies.. Am J Trop Med Hyg.

[44] dos Santos MG, da Silva MFL, Zampieri RA, Lafraia RM, Floeter-Winter LM (2011). Correlation of meta 1 expression with culture stage, cell morphology and infectivity in Leishmania (Leishmania) amazonensis promastigotes.. Mem Inst Oswaldo Cruz.

[45] Uliana SR, Affonso MH, Camargo EP, Floeter-Winter LM (1991). Leishmania: genus identification based on a specific sequence of the 18S ribosomal RNA sequence.. Exp Parasitol.

[46] Sambrook J, Russell DW (2001). Molecular cloning : a laboratory manual..

[47] Sanger F, Nicklen S, Coulson A (1977). DNA sequencing with chain-terminating inhibitors.. Proc Natl Acad Sci U S A.

[48] dos Santos MG, Paes LS, Zampieri RA, da Silva MF, Silber AM (2009). Biochemical characterization of serine transport in Leishmania (Leishmania) amazonensis.. Mol Biochem Parasitol.

