# Supplementary material for: Axenic Leishmania amazonensis Promastigotes Sense both the External and Internal Arginine Pool Distinctly Regulating the Two Transporter-Coding Genes
Source: PLoS One. 2011 Nov 16;6(11):e27818. doi: 10.1371/journal.pone.0027818 (PMC3218042; doi:10.1371/journal.pone.0027818)
Supplement: Material and Methods S1 — Methodological description of genetic complementation of yeast mutant. (DOC) [file pone.0027818.s001.doc]

**Genetic complementation of yeast mutant**

The 4.7 and 5.1 ORF’s of *L. amazonensis AAP3* regions were obtained by PCR using forward primer: ATGAGCAAGCCTAACGAGCC and reverse: CTACACGAAGAAGCTGTAGT. The PCR products were first cloned into pGEM- T (Invitrogen) and then, using appropriated restriction enzymes of plasmid linker, transferred to pYES2. The recombinant plasmids were certified by restriction enzymes analysis and sequencing. The transformation of *S. cerevisiae* was performed using Gietz and Schiestl protocol. The mutant strain GAP1/YHR039W as well as a wild type strain was grown in YPAD medium for overnight culturing. From this first culture, 2.5 x10^8^ cells were inoculated into 50 mL of pre-warmed YPAD fresh medium. At the time cell concentration reached 2 x 10^7^/ mL, the culture was centrifuged and cells were washed in cold sterile water, for two times. After that the cells were resuspended in 1.0 mL of sterile water. Samples containing 100 μL of competent cells were distributed into 1.5 mL microcentrifuges tubes, centrifuged and the supernatant discarded. In parallel, carrier DNA was boiled for 5 min and then 2.0 mg/mL were mixed with 20 ng of plasmid DNA, in the presence of PEG 3350 37.5% (w/v) and LiAc 0.1 M. The DNA mix was added to cells and then incubated for 40 min at 42^o^C. After that, the cells were collected by centrifugation and spread in appropriated selection agarose dishes. For the first selection YPAD medium (-URA) and then for complementation assays, minimum medium supplemented with ammonium sulphate or with L-arginine (1mg/mL) were used. The colonies were observed after 3 days of incubation at 30^o^C.
