# Supplementary figures and images for: Axenic Leishmania amazonensis Promastigotes Sense both the External and Internal Arginine Pool Distinctly Regulating the Two Transporter-Coding Genes
Source: PLoS One. 2011 Nov 16;6(11):e27818. doi: 10.1371/journal.pone.0027818 (PMC3218042; doi:10.1371/journal.pone.0027818)

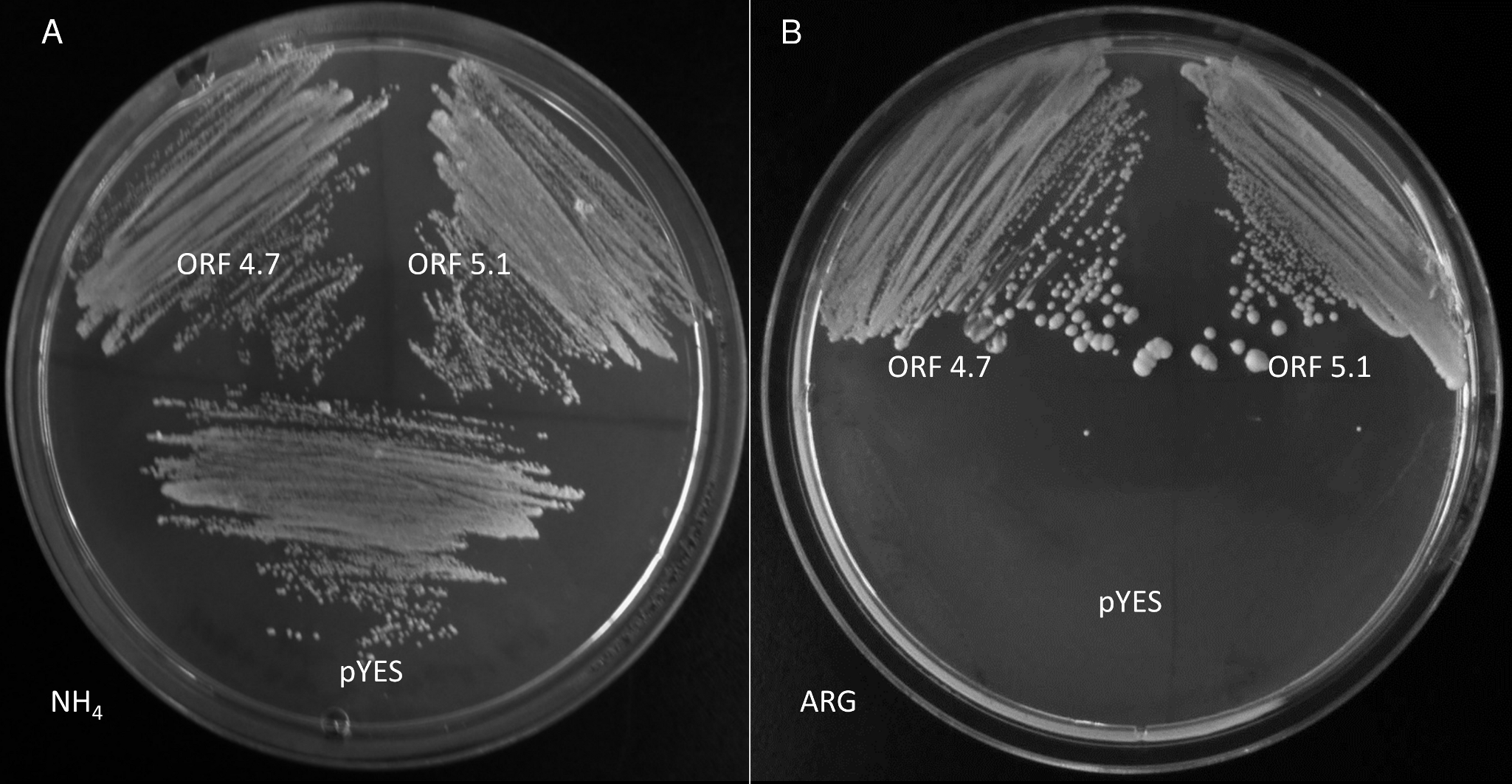

Supplement: Figure S1 — Genetic complementation of yeast mutant certifies the AAP functional character of L. amazonensis amino acid transporter ORFs. pYES2 plasmid DNA carrying 5.1 AAP3 and 4.7 AAP3 ORFs complemented Saccaromyces cerevisiae mutant GAP1/YHR039W. The transformed yeast clones, ORF 4.7 and ORF 5.1, obtained as described in Material and Methods S1 were able to growth in the minimal medium supplemented with 1 mg/m of L-arg (A). The mutant transformed with the recipient plasmid pYES2 alone, only grew in a medium containing ammonium as (NH4+) as nitrogen source (B). (TIF) [file pone.0027818.s002.tif]

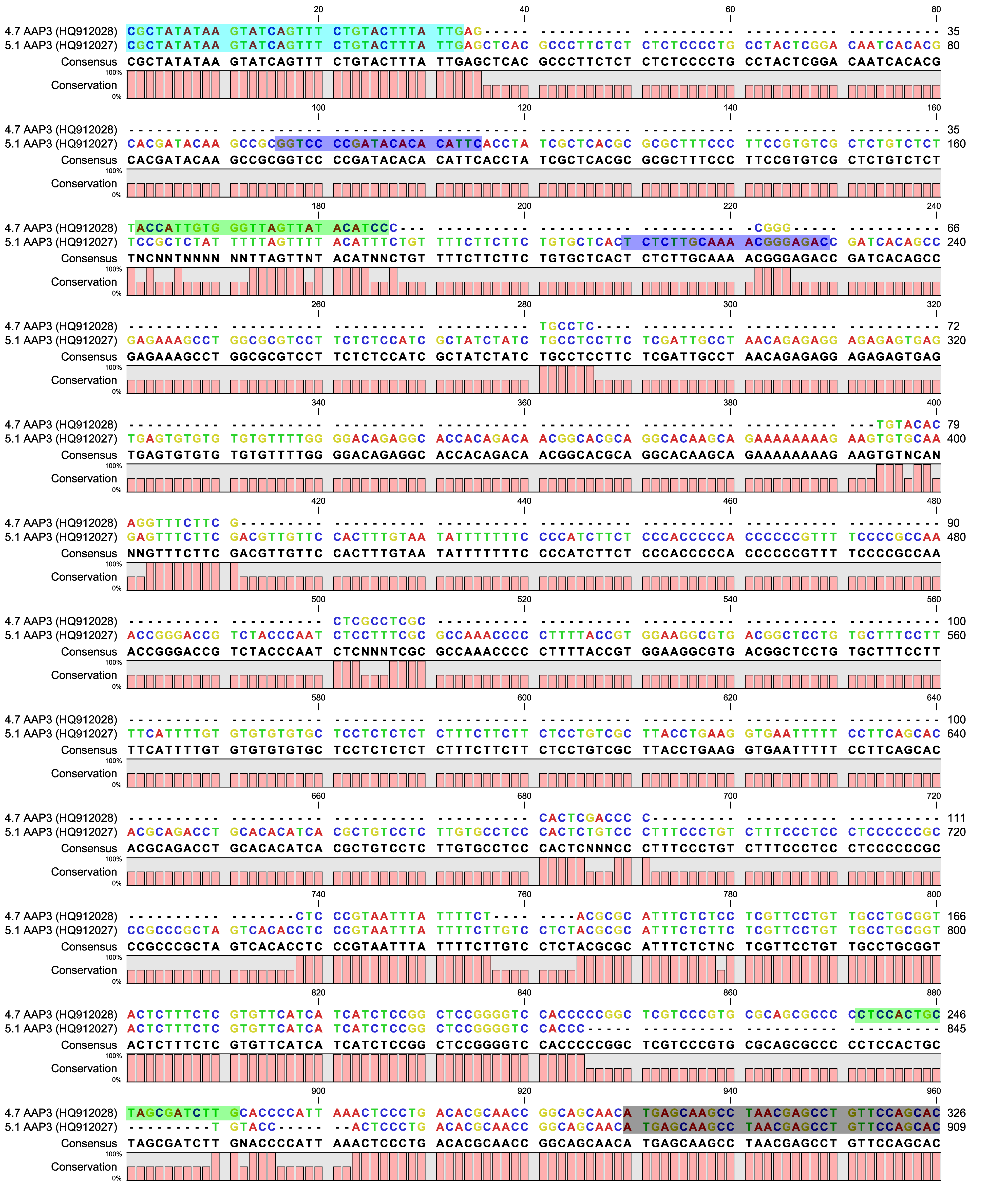

Supplement: Figure S2 — Sequence alignment of the 5′UTRs from 5.1 AAP3 mRNA and 4.7 AAP3 mRNA. Cyan-colored box represents the Spliced-Leader RNA sequence. Gray box represents the ORF beginning. Blue boxes show the primers for amplifying the 5.1 AAP3 mRNA, and the green boxes show the primers for amplifying the 4.7 AAP3 mRNA. (TIF) [file pone.0027818.s003.tif]
